# Biochemical and histopathological evidence for beneficial effects of Empagliflozin pretreatment on acetic acid-induced colitis in rats

**DOI:** 10.1186/s12876-023-02958-2

**Published:** 2023-09-27

**Authors:** Fereshteh Nazari-Khanamiri, Abbas Jafari, Zeinab Esmaeilzadeh, Morteza Ghasemnejad-Berenji

**Affiliations:** 1 School of Pharmacy, Student Research Committee, Urmia University of Medical Sciences, Urmia, Iran; 2 Cellular and Molecular Research Center, Research Institute on Cellular and Molecular Medicine, Urmia University of Medical Sciences, Urmia, Iran; 3 Department of Nutrition, School of Medicine, Urmia University of Medical Sciences, Urmia, Iran; 4 Department of Biochemistry, School of Medicine, Urmia University of Medical Sciences, Urmia, Iran; 5 Experimental and Applied Pharmaceutical Sciences Research Center, Urmia University of Medical Sciences, Urmia, Iran; 6 Department of Pharmacology and Toxicology, School of Pharmacy, Urmia University of Medical Sciences, Urmia, Iran

**Keywords:** Ulcerative colitis, Empagliflozin, Oxidative stress, Acetic acid

## Abstract

**Background:**

Ulcerative Colitis (UC) is a disorder which oxidative stress plays a critical role in its pathogenesis. Empagliflozin (EMPA) is a sodium-glucose cotransporter-2 (SGLT2) inhibitor that has been shown to have anti-inflammatory and antioxidative effects. The aim of this study was to investigate the protective effects of EMPA on acetic acid (AA) induced colitis in rats.

**Methods:**

A total of twenty-four rats were divided into four groups (six animals in each group) as follows: (1) Control group; (2) acetic acid (AA)-induced colitis group (AA); (3) EMPA treatment group (AA + EMPA); (4) Dexamethasone (Dexa) treatment group (AA + Dexa). Animals in pre-treatment groups received EMPA (10 mg/kg, i.p.) or dexamethasone (4 mg/kg, i.p. as reference drug) for four consecutive days before induction of colitis by intra-rectal acetic acid (4% v/v) administration. Twenty-four hours after AA administration, rats were sacrificed and the colon tissues were removed for histopathological and biochemical evaluations.

**Results:**

Pretreatment with EMPA significantly decreased colon weight/length ratio (81.00 ± 5.28 mg/cm vs. 108.80 ± 5.51 mg/cm) as well as, macroscopic (2.50 ± 0.57 vs. 3.75 ± 0.25) and histological scores (3.3 ± 0.14 vs. 1.98 ± 0.14) compared to the AA-induced colitis group (p < 0.01). Pretreatment with EMPA significantly reduced malondialdehyde (MDA) (324.0 ± 15.93 vs. 476.7 ± 32.26 nmol/mg p < 0.001) and increased glutathione level (117.5 ± 4.48 vs. 94.38 ± 3.950 µmol/mg, p < 0.01) in comparison to the AA-induced colitis group. Furthermore, a significant increase in catalase (44.60 ± 4.02 vs.14.59 ± 2.03 U/mg, P < 0.01), superoxide dismutase (283.9 ± 18.11 vs. 156.4 ± 7.92 U/mg, p < 0.001), and glutathione peroxidase (10.38 ± 1.45 vs. 2.508 ± 0.37, p < 0.01) activities were observed by EMPA pretreatment when compared to the AA-induced colitis group. These results were in line with those of the reference drug.

**Conclusions:**

It is concluded that EMPA could effectively reduce the severity of tissue injury in experimental colitis. This protective effect may be related to the antioxidative effects of EMPA drug.

## Background

Ulcerative Colitis (UC) is a chronic inflammatory bowel disease that disrupts the homeostasis of the digestive tract and causes uncontrolled intestinal inflammation [1]. The pathophysiology of UC is not clearly understood but it had shown that several factors such as environment, genetics, autoimmunity, and intestinal microbiota are involved [2]. Various animal models of intestinal inflammation have been established and acetic acid-induced colitis is one of the widely used [3]. In this experimental model, leukocytes, including, lymphocytes, neutrophils, and macrophages, have been indicated to infiltrate inflamed mucosa [4]. Simultaneously, there are various reactive oxygen species in the colonic tissue. Oxidative stress, with its effects on enhanced lipid peroxidation and free radical generation, is the base of colitis evolution [5]. The over-production and sudden release of reactive oxygen species (ROS) by immune cells have an essential role in the pathophysiology of UC [6]. Although many treatments have been proposed and clinically proven, many patients do not respond to the currently available options. Common treatments demonstrate significant side effects on prolonged use [7]. Empagliflozin (EMPA) is a sodium-glucose cotransporter 2 (SGLT2) inhibitor, and it is an anti-diabetic drug. SGLT2 locates in proximal tubes in the kidneys and reduces glucose levels by inhibiting glucose reabsorption [8]. EMPA is a new class of antidiabetic drugs [9]. In addition to its antidiabetic properties, EMPA was reported to reduce diabetic renal and cardiovascular complications in both human and animal studies [10–12]. Investigations reported the potential anti-inflammatory, anti-oxidative and anti-apoptotic properties of EMPA which could also exert possible beneficial effects on colitis [13, 14]. It has been reported that in streptozotocin-induced type 1 diabetic rats EMPA can reduce ROS in pancreatic β-cells [15]. Another study revealed that EMPA can ameliorate oxidative stress in aortic vessels and prevent endothelial dysfunction in the aortic rings of streptozotocin-induced type 1 diabetic rat [16]. Furthermore, it has been shown that EMPA could attenuate transient cerebral ischemia/reperfusion injury in hyperglycemic rats via repressing oxidative stress [17]. Based on these observations and considering the role of oxidative stress in the pathophysiology of acetic-acid (AA)-induced UC, we speculate that pretreatment with EMPA might have a protective effect on AA-induced UC. Therefore, we evaluated the potential protective effects of EMPA on the experimental UC model.

## Methods

### Chemicals

EMPA was purchased from Tehran Chemie Pharmaceuticals (Tehran, Iran). Dexamethasone (Dexa) was obtained from Iran Hormone Pharmaceuticals (Tehran, Iran). Ketamine and Xylazine were bought from Alfasan Co. (Woerden Holland), and analytical chemical compounds such as thiobarbituric acid, acetic acid, formalin solution, and DMSO was purchased from Merck Co. (Darmstadt, Germany). All other chemicals used in the study were of the most purity grade available commercially.

### Animals

Twenty-four male, Wistar Rats (200–220 g) were obtained from the Experimental Animal Laboratory of Urmia University of Medical Sciences, Faculty of Pharmacy, and were randomly housed in appropriate cages under a temperature of 22 ± 2 °C and a 12-h light/ dark cycle (lights on from 6:00 a.m. to 6:00 p.m.), with free access to food and tap water. All the animals were acclimatized to laboratory conditions for a week before the commencement of the experiment. Experimental procedures were approved by the ethical committee of Urmia University of Medical Sciences (IR.UMSU.REC.1400.127). All methods are reported in accordance with ARRIVE guidelines.

### Colitis induction

Rats were fasted overnight and then were anesthetized with an intraperitoneal injection of ketamine (100 mg/kg) and xylazine (10 mg/kg) [18]. Diluted AA (2 ml, 4% v/v in 0.9% saline) was slowly instilled into the rat colons using a soft pediatric catheter (2.7 mm diameter) inserted into the rat anus for 8 cm. After AA administration, rats were held vertically for 2 min (to induce colitis and to prevent AA leakage). Control animals underwent the same procedure using an equal volume of normal saline instead of AA solution [19, 20].

### Experimental design

A total of twenty-four rats were divided into four groups (six animals in each group) as follows: [1] Control group; [2] AA-induced colitis group (AA); [3] EMPA treatment group (AA + EMPA); [4] Dexa treatment group (AA + Dexa). In order to pretreatment in AA + EMPA or AA + Dexa groups, EMPA (10 mg/kg) or Dexa (8 mg/kg) respectively was injected intraperitoneally for 4 consecutive days, and on the fifth day, UC was induced by intra-rectal instillation of AA. Animals in the control and AA groups received intraperitoneal DMSO as a vehicle for 4 days and on day 5 normal saline and AA were instilled into the lumen of the colon respectively. Twenty-four hours after the colitis induction, the animals were sacrificed under deep anesthesia. Harvested colons were opened longitudinally and washed in normal saline after which they were examined [21]. The colon (5-6 cm) specimen was dissected, and washed with saline solution. Then after weighing the tissue, an appropriate photo was taken. Then a small cross-section was fixed in 10% formaldehyde solution for histopathological evaluation. The remaining tissue was kept at − 80°C until analysis. To produce 10% (w/v) homogenate, tissues were homogenized with ice-cold phosphate buffer (0.01 M, pH 7.4). for 5 min by a manual homogenizer. Then the homogenate was centrifuged at 12,000× rpm for 20 min. The clear supernatant was used for the analysis. All procedures were performed at 4^°^C.

### Colon weight-to-length ratio

The abdomens of experimental animals were dissected, and 10 cm of their colons were isolated, rinsed gently with normal saline, and was weighted. The weight-to-length ratio was then calculated by dividing the wet weights by the length of the colon to obtain an index of disease-caused edema and wall thickening. Furthermore, macroscopic scoring was performed.

### Determination of extent of macroscopic damage

The extent of macroscopic damage was evaluated by assigning scores as described by Millar [22]. The damage to colons was based on observed clinical features, and scores were assigned accordingly; no macroscopic alteration (score 0), mucosal erythema only (score 1), mild mucosal edema, slight bleeding or small erosions (score 2), moderate edema, slight bleeding ulcers or erosions (score 3) and severe ulceration, edema and tissue necrosis (score 4). These scores were assigned blindly after careful observation of colons.

### Histological evaluation

Colon tissues were removed under surgery proximately from 2 cm above the anal margin and they were splitting longitudinally, and washed with normal saline. Colon tissues were photographed for macroscopic consideration. Colon tissues were halved. One-half of them were transferred to a -80 centigrade freezer for examining specific oxidative stress markers and one other half was fixed in formalin to prepare the H&E slide. The histological grading was assessed according to previously described criteria [23, 24] as follows: 0, no signs of inflammation; 1, very low level; and 2, low level of leukocyte infiltration; 3, high level of leukocyte infiltration, high vascular density, thickening of the colon wall; and 4, transmural infiltration, loss of goblet cells, high vascular density, thickening of the colon wall.

### Evaluation of antioxidant enzyme activity and oxidative stress biomarkers

Levels of colon tissue oxidative stress markers were measured according to the method described previously [25]. Briefly, an ice-cold solution of phosphate-buffered saline (PBS) (pH 7.4) was used to rinse 1 g of colon tissue for two minutes. The colon tissues were homogenized in 10 mL of ice-cold 50 mM potassium phosphate buffer. The obtained homogenate was centrifuged at 12,000× rpm for 20 min at 4 ^o^ C, and the supernatant was aliquoted for further tests. The activity levels of three antioxidant enzymes catalase (CAT), superoxide dismutase (SOD), and glutathione peroxidase (GPx) were determined by kits, according to the manufacturer’s procedure. The malondialdehyde (MDA) level, which is an indicator of lipid peroxidation, was determined according to the method of Ohkawa et al. with slight modifications [26, 27]. In brief, 0.1 ml of tissue homogenate was added 0.9 ml of 1.8% sodium dodecyl sulfate (SDS), 1.5 ml of 20% acetic acid solution (pH 3.5), and 1.5 ml of aqueous solution of Thio barbituric acid. The mixtures were heated at 95^o^C for 60 min. After cooling with tap water, 5 ml of the mixture of n-butanol and pyridine (15:1, v/v) was added, shaken vigorously, and then centrifuged at 4,000 rpm for 10 min. The organic layer was taken, and its absorbance at 532 nm was measured. 1,1,3,3- Tetramethoxypropane was used as an external standard, and the level of lipid peroxides was expressed as nmol of MDA per gram of wet tissue. Reduced glutathione (GSH), commonly known non-enzymatic antioxidants involved in the detoxification of xenobiotics, was measured as described previously by Hu et al. [28]. In brief, a volume (10 µL) of supernatant was mixed with 200 µL of Tris–EDTA buffer (Tris base [0.25 M], EDTA [20mM], pH 8.2) and 4 µL of DTNB (5,5-dithiobis-2-nitrobenzoic acid) (10 mM) in methanol. Then, the samples were incubated at 37 °C for 30 min to appear yellow color. The absorbance of the supernatant was measured against a blank at 412 nm. Reduced GSH was expressed as nmol/mg protein.

### Statical analysis

Statistical analyses were done using GraphPad Prism software version 6.01 for Windows (GraphPad Software, San Diego Inc., California, USA). Data were expressed as mean ± standard error mean (SEM). One-way analysis of variances followed by Tukey’s post hoc test was conducted to compare the means among the groups. The significance was considered as a P value of less than 0.05.

## Results

### Effect of EMPA on colon weight-to-length ratio

**Fig. 1:**
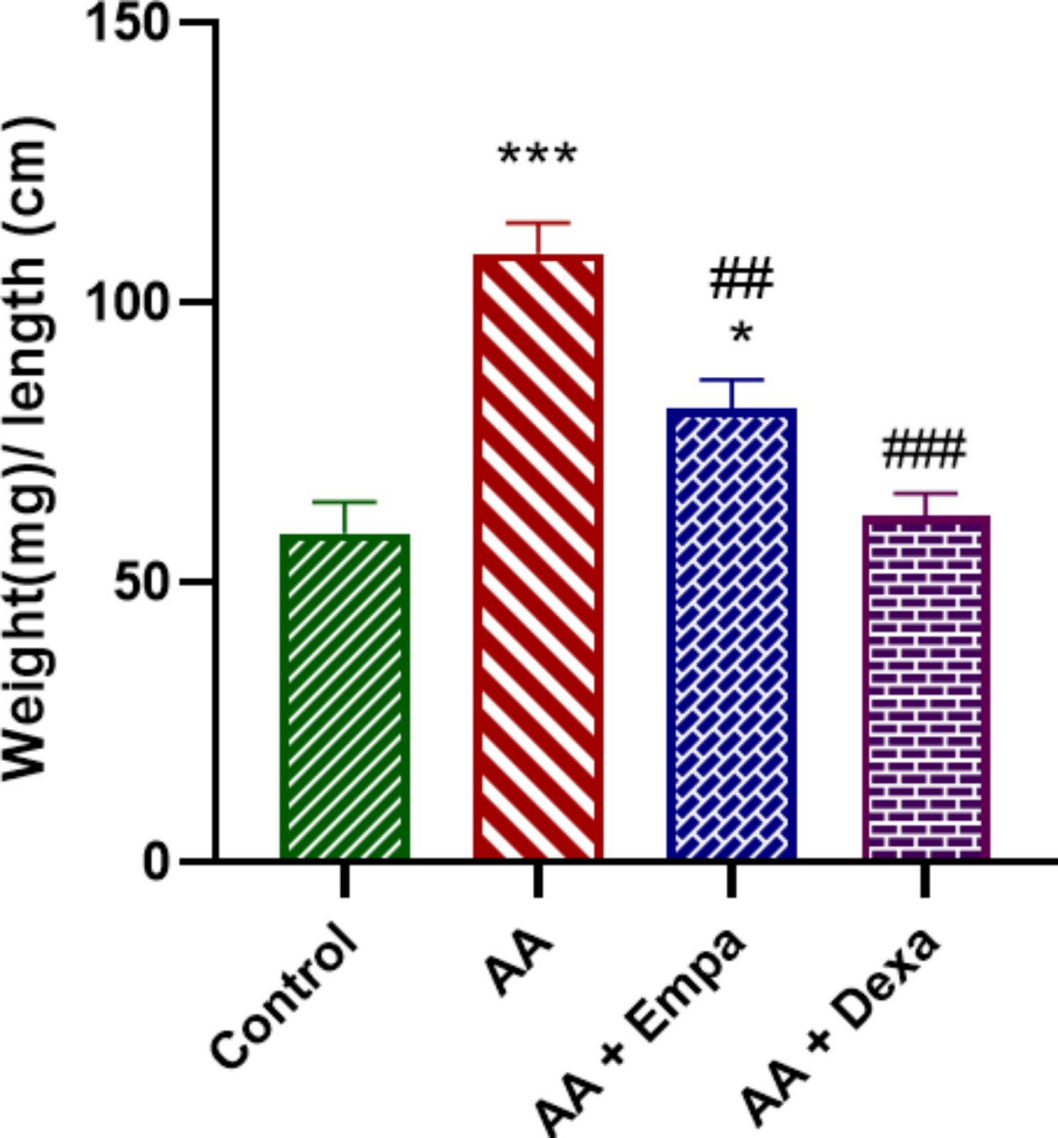
Effect of EMPA on colon weight/length ratio. The results were expressed as mean ± SEM and analyzed by ANOVA followed by Tukey test. *p < 0.05 vs. control group, ***p < 0.001 vs. control group, ^##^p < 0.01 vs. AA group ^###^p < 0.001 vs. AA group. AA: Acetic acid, EMPA: Empagliflozine, Dexa: Dexamethasone

Rat colon weight-to-length (mg/cm) ratio was determined for all groups and the means were determined. The weight-to-length ratio of the control group was 58.80 ± 5.69 mg/cm which increased considerably to 108.80 ± 5.51 mg/cm in the AA group (p < 0.001(compared to control group, (Fig. 1). However, this was reduced to 61.80 ± 4.18 mg/cm in AA + Dexa group (p < 0.001 compared to AA group, Fig. 1). This ratio in AA + EMPA group was 81.00 ± 5.28 mg/cm with was significantly lower than AA group (p < 0.01, Fig. 1).

### Effect of EMPA on macroscopic changes of colitis

**Fig. 2:**
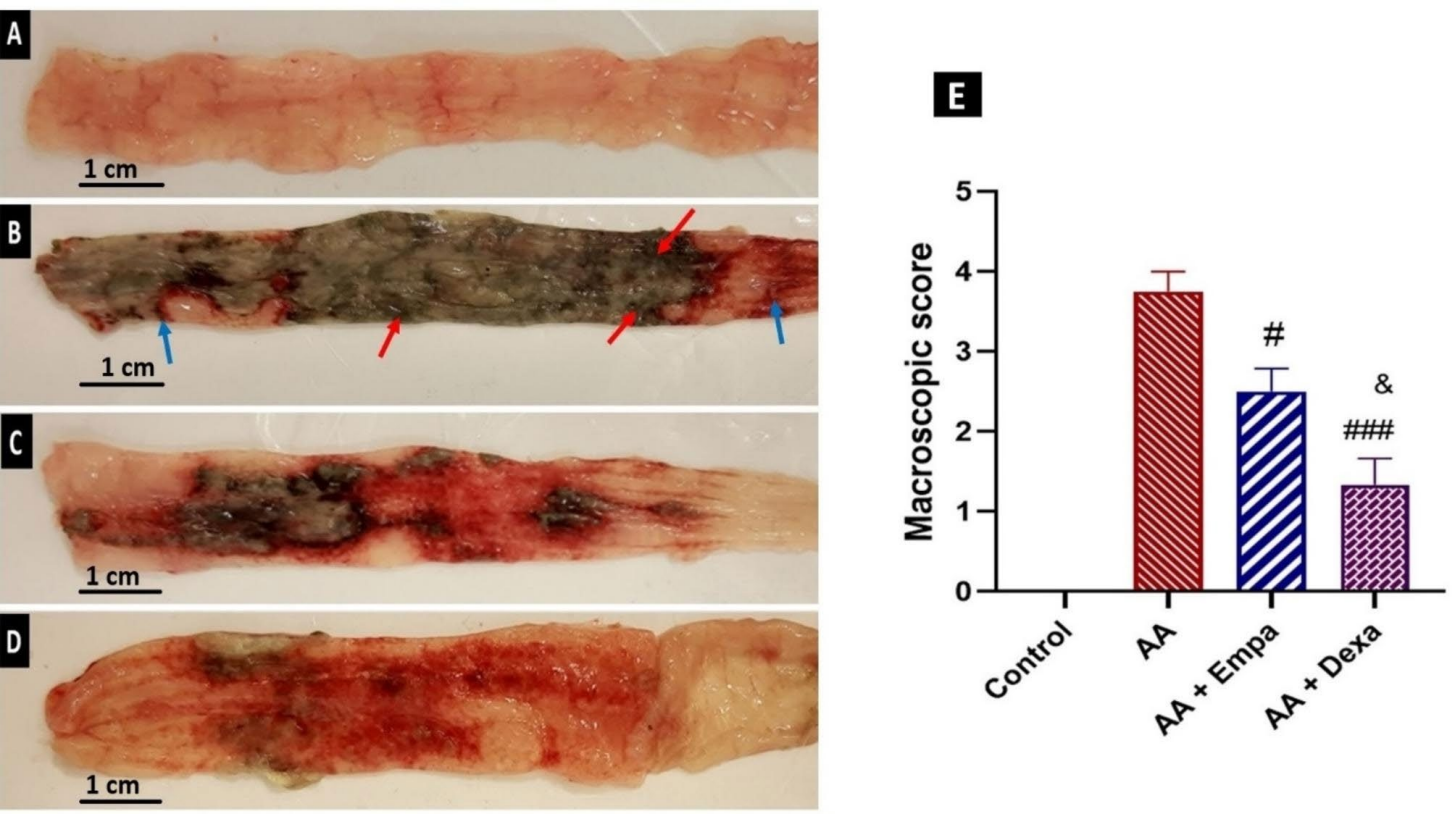
Effects of EMPA and Dexa on colonic macroscopic damage in rats with AA-induced colitis. pretreatment with EMPA or Dexa was done for 4 consecutive days before induction of colitis by AA. Control and AA groups received vehicle in an equal volume. (A) Macroscopic appearance of colonic mucosa of normal rats, showing normal mucosa with intact epithelial surface. (B) Macroscopic appearance of colonic mucosa of AA group showing severe ulceration (blue arrows), and tissue necrosis (red arrows) of the colon tissue. (C) Macroscopic appearance of colonic mucosa of AA + EMPA group. (D) Macroscopic appearance of colonic mucosa of AA + Dexa group (E). Mean scores of the tissue damage observed in colonic specimens of animals in different experimental groups. The results were expressed as mean ± SEM (n = 6). ^#^p < 0.05 vs. AA group; ^###^p < 0.001 vs. AA, ^&^p < 0.05 vs. AA + EMPA group. AA: Acetic acid, EMPA: Empagliflozine, Dexa: Dexamethasone

After assessing colons, the naive control group showed intact mucosa and serosa with no signs of tissue damage or hemorrhage (Fig. 2A). No mortality was observed in animals receiving intracolonic 4% acetic acid (2 mL) solution. Acetic acid caused severe ulceration, hemorrhage and extensive necrosis of colon tissue surface in the AA group (Fig. 2B); Pretreatment with AA + EMPA showed a significant decrease in macroscopic damage compared to the AA group (P < 0.05; Fig. 2C) and Dexamethasone protected against mucosal damage and tissue necrosis and significantly reduced macroscopic damage compared to the AA group (P < 0.001; Fig. 2D). Treatment with EMPA or Dexa attenuated the extent and severity of the morphological alterations associated with AA. Macroscopic scores were assigned to numerically quantify tissue damage. The highest score was recorded in the AA group with an average score of 3.75 ± 0.25 compared to the control group with a score of 0 (Fig. 2E). AA + Dexa group recorded the lowest macroscopic score of 1.33 ± 0.57 (p < 0.001 compared to AA group) while the macroscopic score in AA + EMPA group was 2.50 ± 0.57 (p < 0.05 compared to AA group, Fig. 2E).

### Effect of Empagliflozin on histopathological changes

**Fig. 3:**
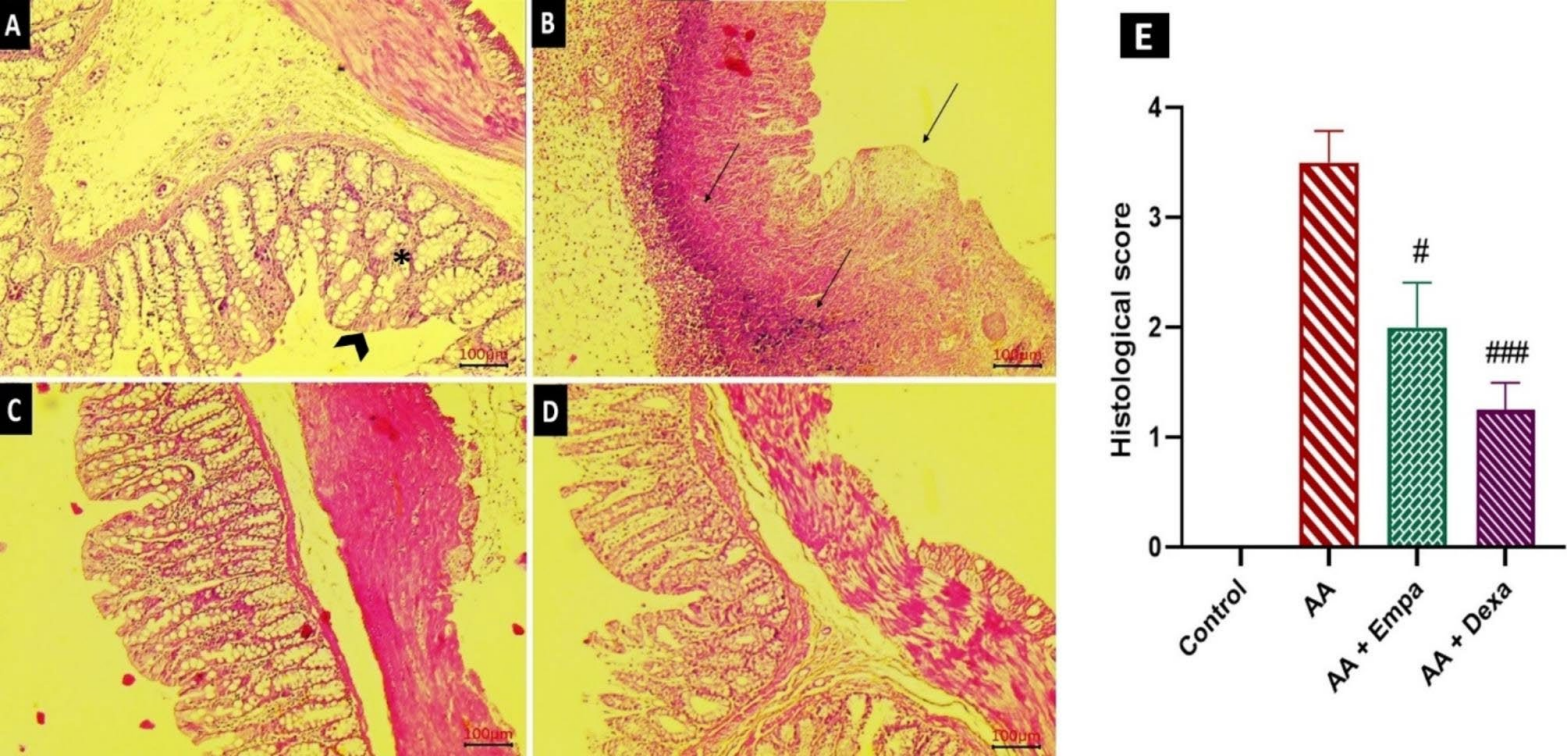
Photomicrographs of section of colons from rats stained with H&E. Colon microscopic image of (A) Normal rat with intact epithelial (head arrow) and Mucosal layer (*); (B) AA-induced colitis rats with extensive damage (black arrows) including ulceration; Hyperemia; Necrosis; edema in submucosa, cellular infiltration, and Cellular hyperplasia (C) Pretreatment with EMPA reduced histological alterations induced by AA (4%) in rat colon. (D) Dexa treated rats. Magnification 100×, scale bar 100 μm. (E) Represents the altered pathological scores after induction of UC as compared to AA group and its restoration by EMPA and Dexa treatment. The results were expressed as mean ± SEM (n = 6). ^#^p < 0.05 vs. AA group; ^###^p < 0.001 vs. AA group. AA: Acetic acid, EMPA: Empagliflozine, Dexa: Dexamethasone

Microscopic evaluation of hematoxylin and eosin-stained (H&E, Magnification 100×) sections of colonic tissues of normal rats indicated intact epithelium and mucosal layer (Fig. 3A). In contrast, AA-induced UC showed focal crypt distortions and abscesses necrotic destruction of surface epithelium, intense cellular infiltration, and hyperemia of submucosal capillaries (Fig. 3B). Normal mucosal epithelial cells with few colonic lesions were observed in AA + Dexa group (Fig. 3D). Significantly, treatment with AA + EMPA indicated remarkable recovery of the colonic tissue compared to the AA-induced colitis damage (Fig. 3C). The histopathological scores showed noticeably higher tissue damage and inflammation in the colon of the AA group relative to those of the control and AA + Dexa groups, while pretreatment with EMPA reduced the microscopic injury score compared with the AA group (p < 0.05, Fig. 3E).

### Effect of EMPA on antioxidant enzyme activity and oxidative stress biomarkers

**Fig. 4:**
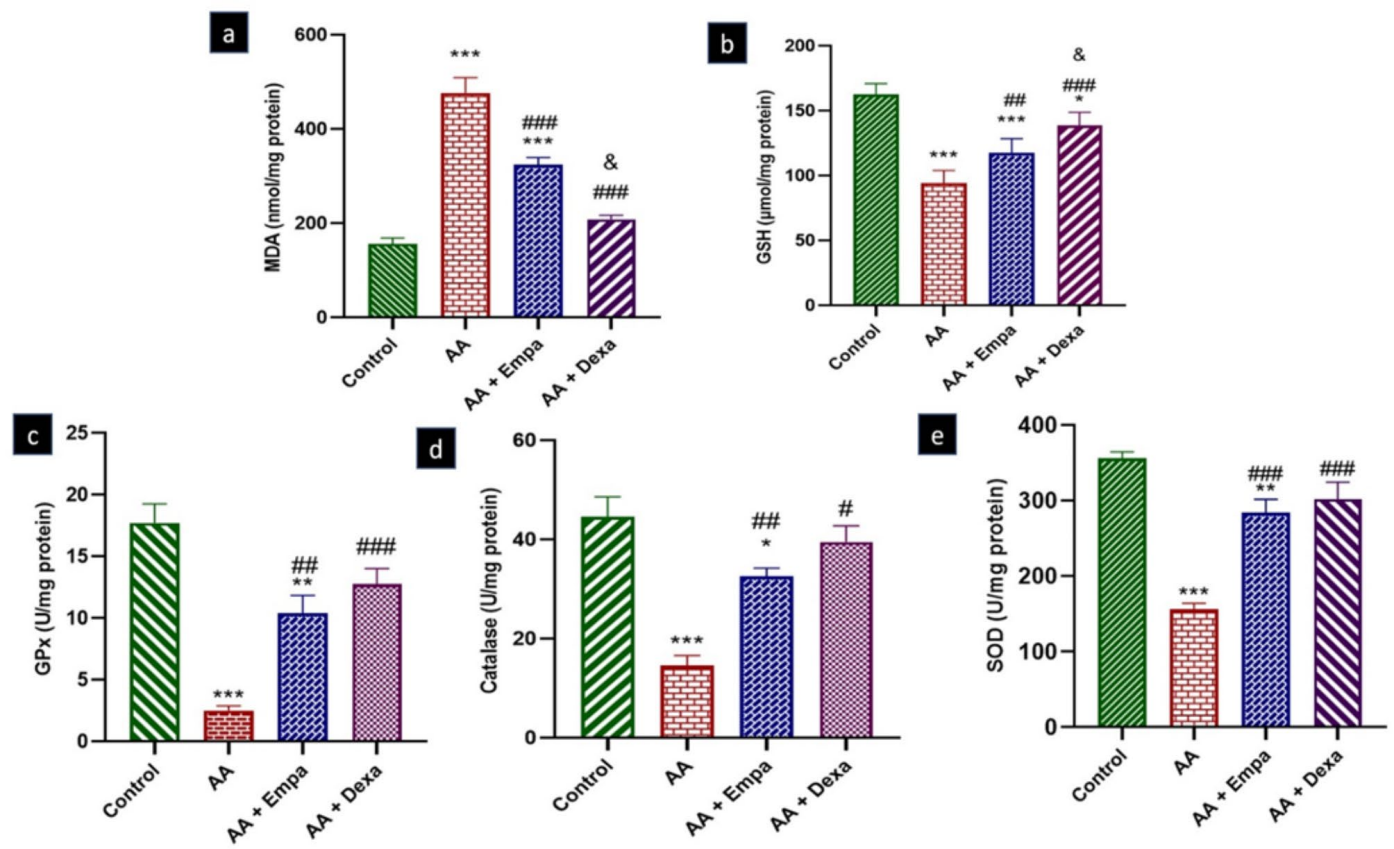
(a) MDA, (b) GSH, (C) GPx, (d) catalase and (e) SOD concentrations in colonic tissue samples from the different experimental groups. Data are expressed as mean ± SEM (n = 6). The results were expressed as mean ± SEM (n = 6). *p < 0.05 vs. control group, **p < 0.01 vs. control group, ***p < 0.001 vs. control group, ^#^p < 0.05 vs. AA group; ^##^p < 0.01 vs. AA group, ^###^p < 0.001 vs. AA group, &p < 0.05 vs. AA + EMPA group. AA: Acetic acid, EMPA: Empagliflozine, Dexa: Dexamethasone

MDA level was significantly increased in AA group (476.7 ± 32.26 nmol/mg protein) versus control group (156.8 ± 12.06 nmol/mg protein) (P < 0.001). MDA level in AA + EMPA and AA + Dexa groups were 324.0 ± 15.93 and 208.6 ± 8.48 nmol/mg respectively which were significantly lower than AA group 476.7 ± 32.26 nmol/mg (P < 0.001; Fig. 4a). The GSH level was significantly decreased in AA group (94.38 ± 3.950 µmol/mg) compared to the control group (162.6 ± 3.358 µmol/mg) (p < 0.001). The GSH level in the AA + EMPA and AA + Dexa groups were 117.5 ± 4.48 and 138.7 ± 5.76 µmol/mg respectively which were significantly higher than in AA group (p < 0.01 and, p < 0.001 respectively; Fig. 4b). There were significant decreases in GPx, CAT and SOD activities in AA group (2.508 ± 0.37, 14.59 ± 2.03 and 156.4 ± 7.92 U/mg protein respectively) versus control group (17.72 ± 1.542, 44.60 ± 4.02 and 356.6 ± 8.38U/mg protein respectively). These values in AA + EMPA treated group (10.38 ± 1.45, 32.55 ± 1.74 and 283.9 ± 18.11 U/mg protein respectively) were also significantly higher than those in AA group. However, there were no significant differences in the value of these parameters between AA + EMPA and AA + Dexa groups (Fig. 4c, d, e respectively).

## Discussion

The present study demonstrates that EMPA a new anti-diabetic agent, reduces tissue damage in an experimental model of AA–induced colitis. AA-induced colitis has been widely used for evaluating the efficacy of drugs effective in UC and mimics this condition in humans [29]. In present study, treatment with EMPA markedly inhibited mucosal hyperemia, ulcer formation and produced reduction in the weight/length ratio of the colon [30].Different factors such as increased production of inflammatory mediators, enhanced vaso-permeability, prolonged neutrophil infiltration, and the induction of oxidative stress are present in this experimental model [5, 30]. Although the etiology of UC remains unknown, the role of oxidative stress in its pathogenesis is well established. A number of experimental and clinical studies indicate towards the role of oxidative stress both in the induction and perpetuation of UC. In the present study, excessive generation of free radicals was indicated by the altered levels of oxidative stress markers and antioxidant defense following intra-colonic instillation of AA in experimental animals. Lower levels of GSH, a tripeptide reducing agent that scavenges free radicals, and higher levels of MDA, a measure of lipid peroxidation, were observed in the colonic tissue of AA group as compared to control group. The activities of enzymatic antioxidants CAT, GPx and SOD were also decreased following induction of colitis. In our study, Dexa was used as a reference drug to delineate the efficacy of a test substance. The wet weight of the colon tissue is considered a reliable and sensitive indicator of the severity and extent of the inflammatory response [31]. In the present study, pre-treatment with EMPA in the AA-induced colitis significantly decreased the wet weight/length ratio of colon. In line with this our histological evaluations showed that pretreatment with EMPA ameliorates the histological damages induced by AA. Oxidative stress has the main role in the pathogenesis of AA-induced colitis [32]. Oxidative stress is an imbalance between ROS generation and antioxidant defense mechanisms. It is believed to be the definite underlying cause that leads to cellular dysfunction [27, 33]. To assess oxidative state in this study, we measured GSH, MDA content, and CAT, SOD, and GPx activities in colon homogenates. According to our results it was revealed that intrarectal administration of AA leads to a significant deterioration in the levels of these parameters (lowering in GSH content, and CAT, SOD, and GPx activities, while MDA content showed an elevation) where these results are consistent with results of previous studies which has been shown that AA-induced colitis leads to an extensive increase in the production of oxidative free radicals such as ROS [34, 35]. The sudden enhancement of tissue ROS triggers abnormal vascular endothelium, inflammation, and increased expression of inflammatory cytokines and induces extensive tissue injury [36–38], whereas oxidative free radicals induce the degradation of membrane-bound phospholipids and the destruction of structural proteins [39]. Our results showed that pretreatment with EMPA significantly reduces the level of MDA, in comparison to the AA group. SOD, CAT and, GPx are common antioxidant enzymes that were evaluated in this study in order to clarify the potency and efficacy of EMPA in ameliorating oxidative stress. Related to the results of the current study, EMPA increased the level of SOD compared to the AA group however there was no significant difference in increasing SOD in the pretreatment of EMPA compared to DEXA therapy. This shows the potential benefits of EMPA in stress oxidative inhibition. According to our results, it was revealed that the CAT activity in EMPA group was significantly higher than AA group. In addition to these results, it was indicated that EMPA pretreatment markedly increased GSH as a non-enzymatic antioxidant marker. In a similar effect, EMPA increases the level of GPx as a catalyzing enzyme that reduces peroxide radicals to oxygen and alcohol. In our study, the highest level of GPx was observed in the control group and pretreatment of EMPA significantly increased the amount of GPx compared to the AA group. There were no significant differences between EMPA and DEXA pretreated groups in increasing GPx. These results are confirmed by previous studies that revealed the anti-oxidant effect of EMPA in cerebral ischemia/reperfusion injury in hyperglycemic rats [17], and ethanol-induced liver injury in mice [40]. Furthermore, it has been reported that EMPA represents renal and myocardial protection which are related to its antioxidant and anti-inflammatory effects [30, 31]. In line with these studies results of current research propose that the mechanisms underlying the ameliorative effects of EMPA on AA-induced colitis can account for the prevention of oxidative stress in the rat colon. Although this association is logical, it should be considered that the reduction of oxidative stress could reduce the inflammatory responses in colon tissue. A previous study has reported that EMPA upregulated Nrf2/HO-1-mediated antioxidant responses in the hearts of C57BL/6J mice treated with a high-fat diet [41]. It’s possible that the observed protective effect of EMPA in our study be related to the activation of this signaling pathway. However, we didn’t evaluate the direct effect of EMPA on Nrf2/HO-1 and it was one of the limitations of our study. We suggest that in future studies the effect of EMPA on Nrf2/HO-1 will be evaluated.

## Conclusions

In conclusion, the present study revealed that EMPA protects AA-induced ulcerative colitis by inhibiting oxidative biomarkers. Finally, our results may pose promising outcomes for future clinical usage of EMPA as a new safe drug in colitis.

## Data Availability

The datasets generated and/or analyzed during the current study are not publicly available due to dissatisfaction of some authors but are available from the corresponding author on reasonable request.
